# Graves’ Disease Patients with Persistent Hyperthyroidism and Diffuse Lymphoplasmacytic Infiltration in the Thyroid Show No Histopathological Compatibility with IgG4-Related Disease

**DOI:** 10.1371/journal.pone.0134143

**Published:** 2015-07-28

**Authors:** Eijun Nishihara, Mitsuyoshi Hirokawa, Mitsuru Ito, Shuji Fukata, Hirotoshi Nakamura, Nobuyuki Amino, Akira Miyauchi

**Affiliations:** Center for Excellence in Thyroid Care, Kuma Hospital, Kobe, Japan; University of Birmingham, UNITED KINGDOM

## Abstract

**Background:**

IgG4-related disease is a novel disease entity characterized by diffuse lymphoplasmacytic infiltration rich in IgG4-positive plasma cells and fibrosis into multiple organs. There is still controversy over whether some thyroid diseases are actually IgG4-related disease. The objective of this study was to elucidate the clinicopathological features of Graves’ disease with diffuse lymphoplasmacytic infiltration in the thyroid.

**Patients and Methods:**

Among 1,484 Graves’ disease patients who underwent thyroidectomy, we examined their histopathological findings including the degree of lymphoplasmacytic and fibrotic infiltration and levels of IgG4-positive plasma cells in the thyroid. Their clinical pictures were defined by laboratory and ultrasonographic evaluation.

**Results:**

A total of 11 patients (0.74%) showed diffuse lymphoplasmacytic infiltration in the stroma of the thyroid gland. Meanwhile, other patients showed variable lymphoid infiltration ranging from absent to focally dense but no aggregation of plasma cells in the thyroid gland. Based on the diagnostic criteria of IgG4-related disease, 5 of the 11 subjects had specifically increased levels of IgG4-positive plasma cells in the thyroid. Fibrotic infiltration was present in only 1 patient developing hypothyroidism after anti-thyroid drug treatment for 4 years, but not in the other 10 patients with persistent hyperthyroidism. Obliterative phlebitis was not identified in any of the 11 subjects. Thyroid ultrasound examination showed 1 patient developing hypothyroidism who had diffuse hypoechogenicity, but the other hyperthyroid patients had a coarse echo texture.

**Conclusions:**

In our study, Graves’ disease patients with persistent hyperthyroidism who had diffuse lymphoplasmacytic infiltration rich in IgG4-positive plasma cells in the thyroid showed no concomitant fibrosis or obliterative phlebitis.

## Introduction

IgG4-related disease is a novel disease entity characterized by diffuse lymphoplasmacytic infiltration rich in IgG4-positive plasma cells into multiple organs. Concomitant fibrosis and obliterative phlebitis are usually detected around IgG4-positive plasma cells. An elevated concentration of serum IgG4 (beyond 135 mg/dL) is helpful to identify IgG4-related disease before histopathological examination from biopsy or surgical specimens [1]. The etiology of IgG4-related disease is still unknown, while the progression of cellular infiltration or fibrosis causes enlargement or dysfunction of the affected organ.

Among autoimmune thyroid diseases, diffuse lymphoplasmacytic infiltration is the most characteristic feature of Hashimoto’s thyroiditis, in which the follicular epithelium can be quite scant in areas of intense lymphoplasmacytic infiltration [2]. Plasma cells detected in Hashimoto’s thyroiditis show polyclonality with staining for IgG, IgM, and IgA heavy chains and kappa and lamda light chains [3]. Furthermore, Hashimoto’s thyroiditis is classified into several subtypes that present with distinct clinicopathological features. A new subtype of Hashimoto’s thyroiditis shows histopathological findings that are indistinguishable from those of IgG4-related disease, which is referred to as IgG4 thyroiditis [4,5]. These histopathological findings of IgG4 thyroiditis have been identified in Graves’ disease patients who rapidly developed hypothyroidism after anti-thyroid drug treatment for 4–7 years [6,7]. Furthermore, elevated serum IgG4 levels (beyond 135 mg/dL) are detected in 6.4% of all Graves’ disease patients [8] and serum IgG4 levels are significantly higher in patients with than without Graves’ ophthalmopathy [9], suggesting that a part of Graves’ disease may overlap with the disease entity of IgG4 thyroiditis or IgG4-related disease.

Here, we screened for the degree of lymphoplasmacytic infiltration using thyroid specimens of Graves’ disease patients. Levels of IgG4-positive plasma cells and further clinicopathological features were evaluated among subjects with diffuse lymphoplasmacytic infiltration.

## Patients and Methods

### Patients

From 2004 through 2012, a total of 1,647 patients with Graves’ disease (313 men and 1,334 women; aged 37 ± 10.5 years (median ± quartile deviation); 11–87 years (range)) underwent total or near-total thyroidectomy at Kuma Hospital. The diagnosis of Graves’ disease was based on the presence of hyperthyroidism, positive thyroid stimulating antibody (TRAb), and increased radioiodine uptake by the thyroid. Among them, 163 patients were excluded due to the main reason for surgical resection of accompanied thyroid tumors. Consequently, we examined the degree of lymphoplasmacytic infiltration in the stroma and other histopathological findings using the thyroid specimens of 1,484 patients. The present study was approved by the ethics committee of Kuma Hospital, and written informed consent was obtained from all the adult subjects as well as the next of kin on behalf of minors for the use of samples for research purposes and for publication of accompanying images. A copy of the written consent is available for review upon requests.

### Laboratory and ultrasonographic evaluation

Concentrations of serum TSH, free triiodothyronine (FT3), and free thyroxine (FT4) were measured with a chemiluminescent immunoassay (Architect i2000, Abbot Japan, Tokyo, Japan). The reference ranges used for serum TSH, FT3, and FT4 were 0.30–5.00 μIU/mL, 1.70–3.70 pg/mL, and 0.70–1.60 ng/dL, respectively. Serum TRAb were measured with a second-generation enzyme-linked immunoassay (RSR Ltd., Cardiff, UK) through July 2008 and with a third-generation electro-chemiluminescent immunoassay (Roche Diagnostic, Mannheim, Germany) from August 2008 through December 2012. The upper cut-off limits of values of the second- and third-generation TRAb are 15% and 1.9 IU/L, respectively. Serum anti-thyroglobulin (TgAb) and anti-thyroid peroxidase (TPOAb) antibodies were measured with commercially available hemagglutination methods (TGHA: Thyroid test and MCHA: Microsome test, Fuji Rebio Inc., Tokyo, Japan, normal ranges less than 100, respectively) or with an electro-chemiluminescent immunoassay (ECLusys Anti-Tg and ECLusys Anti-TPO; Roche Diagnostic, Mannheim, Germany; the upper cut-off limits of values of TgAb and TPOAb were 39.9 U/mL and 27.9 U/mL, respectively). Serum IgG4 levels were measured by nephelometry using IgG subclass (BS-NIA) kits (SRL, Tokyo, Japan). Ultrasonographic examinations were performed as previously described [5,10].

### Histopathological evaluation and immunohistochemistry

After surgical resection, thyroid tissues were routinely fixed in 10% neutral buffered formalin and specimens were embedded in paraffin. Serial sections (4 μm thick) were cut from each paraffin block. For light-microscopic examination, the sections were stained with hematoxylin-eosin (HE). All HE sections of the 1,484 surgical specimens were reviewed by a pathologist (M.H.) to evaluate the histopathological findings, including the levels of lymphoid and plasmacytic cells. The severities of stromal fibrotic changes and obliterative phlebitis were examined and expressed as 3+, severe; 2+, moderate; 1+, mild;-, negative.

Immunostaining for IgG4 (mouse monoclonal, MCO11, Binding Site, Birmingham, UK) and IgG (rabbit polyclonal, A0423, Dako Cytomation, Glostrup, Denmark) was performed following a previously reported method [5]. In 3 high-power fields in each section, immune-positive cells were counted following a previously reported method [11]. Average numbers of IgG4- and IgG-positive cells and their ratio were calculated in each case (Table 1).

**Table 1:** Levels of IgG4-positive plasma cells and concomitant histopathological findings of thyroid specimens from 11 Graves’ patients with diffuse lymphoplasmacytic infiltration.

Cases	Sex	Histopathological findings		Immunohistochemical findings	
		Fibrosis	Obliterative phlebitis	IgG4/HPF	IgG4/IgG (%)
(1)	F	2+	-	155±41	47±2
(2)	M	-	-	70±27	41±6
(3)	F	-	-	68±30	58±11
(4)	F	-	-	47±17	40±8
(5)	F	-	-	38±8	44±5
(6)	F	-	-	101±14	38±5
(7)	F	-	-	73±37	34±3
(8)	F	-	-	49±4	29±8
(9)	F	-	-	21±3	9±3
(10)	M	-	-	9±6	8±6
(11)	F	-	-	7±3	4±0.4

HPF: high power field

### Statistical analysis

The age at surgery, duration of Graves’ disease, levels of TRAb, TgAb, and TPOAb, and weight of the resected thyroid tissue were compared between the subjects and controls employing the Student’s t-test. *P*<0.05 was accepted as indicating significance.

## Results

**Fig 1:**
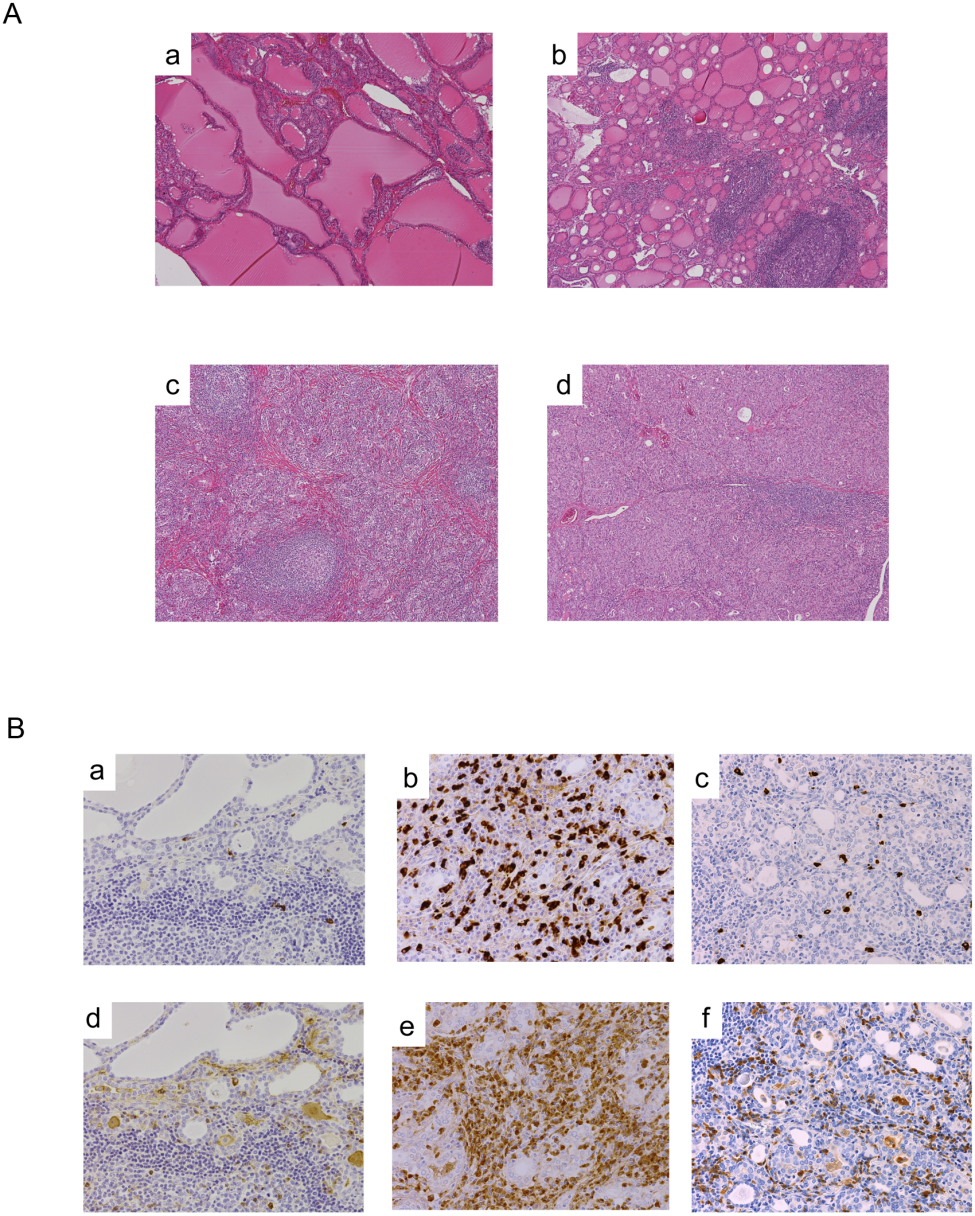
Representative histopathological and immunohistochemical findings of patients with Graves’ disease. (A) Histopathological examination of this thyroid specimen shows follicular swelling, papillary projection, vacuolated colloid, and lymphocytic infiltration and lymphoid follicle formation in the stroma of controls (a and b). Small thyroid follicles, diffuse lymphoplasmacytic infiltration, and Hürtle cell changes are shown in specimens of the subjects (c and d). Stromal fibrotic changes are shown along with lymphoplasmacytic infiltration (c). Case 1: c. Case 2: d. Hematoxylin and eosin-stained, X40. (B) Immunostaining of IgG4 (a, b, c) and of IgG (d, e, f), X40. IgG4- and IgG-positive cells are rarely identified in the control group (a and d). Diffuse infiltration of IgG4-positive cells is shown (b and c). Case 1: b and d. Case 9: c and f.

Among the 1,484 patients with Graves’ disease, 11 subjects (0.74%) showed diffuse lymphoplasmacytic infiltration in the stroma of the thyroid gland (Table 1). Further histopathological features in all 11 subjects were such as marked follicular cell degeneration and oxyphilic change of follicular epithelium (Hürthle cells) (Fig 1A c and d), consistent with Hashimoto’s thyroiditis. Fibrotic infiltration was present in only 1 patient developing hypothyroidism after anti-thyroid drug treatment for 4 years (Fig 1A c) [6], but not in the other 10 patients with persistent hyperthyroidism (Table 1). Obliterative phlebitis was not identified in any of the 11 subjects (Table 1). Immunological analysis of thyroid specimens from the 11 subjects showed that 9 patients had more than 20 IgG4-positive plasma cells per high-power field and 5 patients had a more than 40% IgG4/IgG rate (Table 2, Fig 1B b, c, and e). In contrast, histopathological findings in the other Graves’ disease patients showed diffuse hyperplasia of follicular epithelium and variable lymphocytic infiltration ranging from absent or scant to focally dense with germinal centers, but lacked the plasmacytic aggregation or Hürthle cells (Fig 1A a and b). IgG- or IgG4-positive cells were scarcely detected (Fig 1B a and d).

**Table 2:** Clinical features of Graves’ patients with or without plasmacytic infiltration in the thyroid.

Cases	Age [years]	Duration [years]	Therapy before surgery [/day]	TRAb [IU/L, (%)]	TgAb [U/mL, (fold)]	TPOAb [U/mL, (fold)]	US	TW [g]
(1)	53	5	LT4 75 μg	75.3	>4,000	>600	diffuse low	282
(2)	63	29	MMI 20 mg + KI 100 mg	28.9	>4,000	>600	coarse	155
(3)	56	3	MMI 20 mg + KI 50 mg	(91.8)	(1:409,600)	(1:102,400)	coarse	160
(4)	15	5	KI 100 mg	(85.8)	(1:400)	(1:25,600)	coarse	51
(5)	27	10	PTU 600 mg	(61.7)	(-)	(1:6,400)	coarse	72
(6)	16	6	MMI 30 mg + LT4 75 μg	(92.4)	(1:25,600)	(1:25,600)	coarse	73
(7)	29	7	MMI 40 mg	(95.8)	(1:25,600)	(1:409,600)	coarse	125
(8)	64	7	MMI 20 mg + KI 100 mg	254	>4,000	>600	coarse	267
(9)	25	13	MMI 20 mg + LT4 75 μg	(52.3)	(1:400)	(1:6,400)	coarse	58
(10)	23	14	MMI 15 mg	>400	85.7	>600	coarse	166
(11)	28	0	KI 50 mg	144	538	>600	coarse	26
Controls [n = 80]	35	6	MMI 5–70 mg PTU 50–600 mg KI 50–100 mg	23.4^#^ (84.7)	107.3^#^ (-)^#^	310.4^#^ (1:40) ^#^	Coarse [in all]	123.5

In 80 controls, TRAb, TgAb, and TPOAb were measured using 2 different assay methods, respectively. The values in parentheses indicate the median of percentages using second-generation TRAb and titers using hemagglutination methods for TgAb and TPOAb.

LT4, levothyroxine; MMI, thiamazole; PTU, propylthiouracil; KI, potassium iodide; US, ultrasonographic findings; TW, thyroid weight.

^#^,*P*<0.01

**Fig 2:**
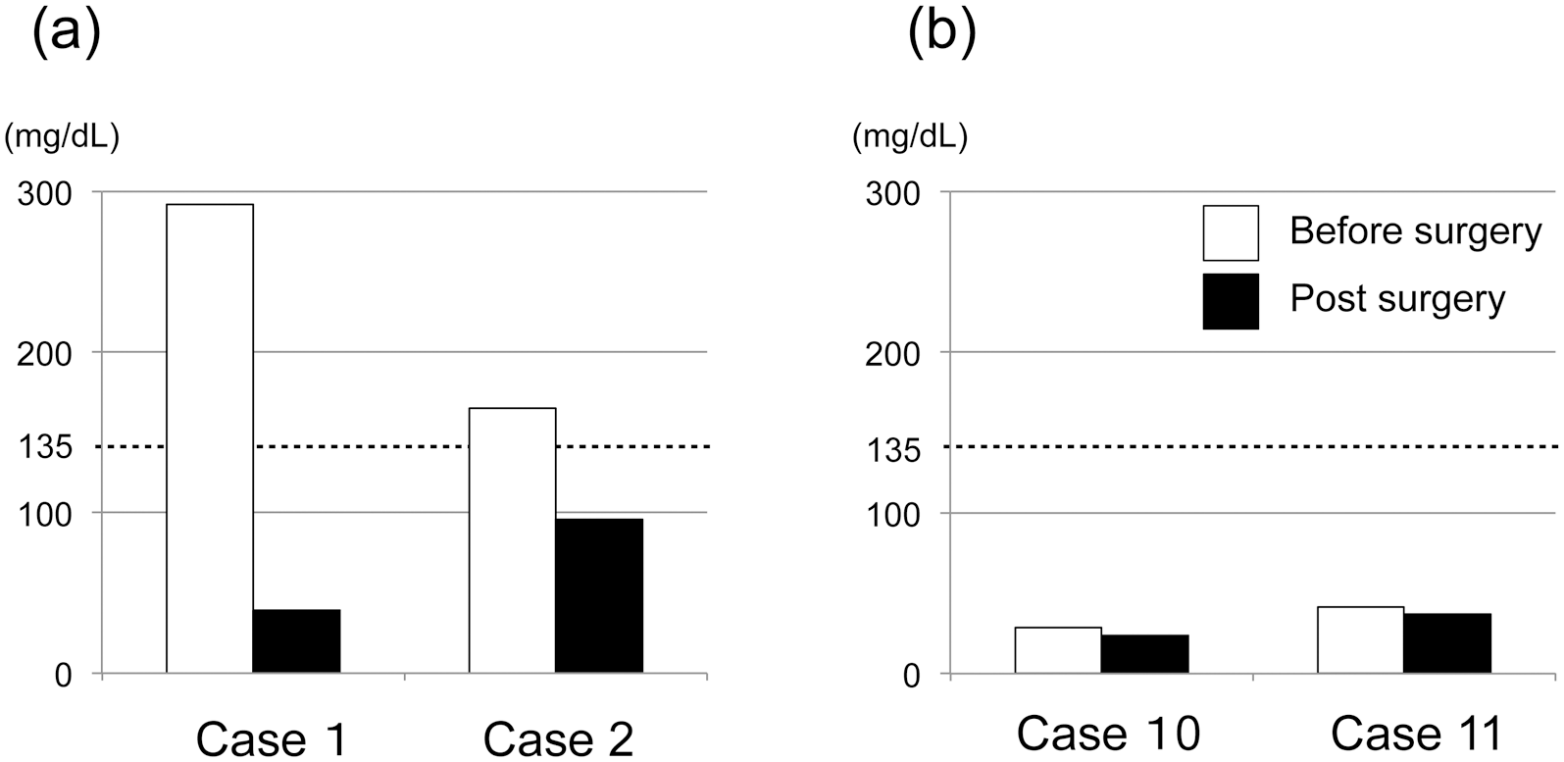
Serum IgG4 levels of subjects before and after thyroidectomy.

Serum IgG4 measurement results before and after thyroidectomy were available for 4 of the 11 patients. Two patients who presented with more than 135 mg/dL serum IgG4 before thyroidectomy had higher levels of IgG4-positive plasma cells in the thyroid (Fig 2a and Table 1). Levels of serum IgG4 decreased to normal values to 3 (case 1) or 14 (case 2) months after thyroidectomy. In the remaining 2 patients, levels of serum IgG4 were within normal ranges before and after thyroidectomy, and their immunopathological findings also showed lower levels of IgG4-positive plasma cells (Fig 2b and Table 1).

To compare several clinical findings between the 11 subjects with diffuse plasmacytic infiltration and controls lacking plasmacytic aggregation, 80 patients were enrolled as a control group by the consecutive extraction of 40 patients from January 2007 and by that of 40 patients from January 2011 (14 men and 66 women; aged 35 ± 7 years). Titers of third-generation TRAb, TgAb, and TPOAb were significantly elevated in the 11 subjects, but the age at surgery, duration of Graves’ disease, weight of the resected thyroid tissue, and levels of second-generation TRAb showed no significant difference between the subjects and controls (Table 2). Thyroid ultrasonographic examination showed that 1 patient (case 1) developing hypothyroidism exhibited diffuse hypoechogenicity, but the other hyperthyroid patients and 80 control patients had a coarse echo texture.

## Discussion

In the spectrum of autoimmune thyroid diseases, several histopathological and immunological findings overlap between Graves’ disease and Hashimoto’s thyroiditis [12,13]. In this study, we identified a small portion (0.74%: 11/1,484 subjects) of Graves’ disease patients who presented with diffuse lymphoplasmacytic infiltration and Hürthle cells in the thyroid. These histopathologilcal findings were indistinguishable from Hashimoto’s thyroiditis [2], and the subjects had significantly higher titers of TgAb and TPOAb than controls (Table 2). In contrast, other Graves’ disease patients presented with varying degrees of lymphocytic infiltration but lacked plasmacytic aggregation in the thyroid (Fig 1A a and b). Although plasma cells are abundantly present in the thyroid tissue of untreated patients with newly diagnosed Graves’ disease, they are rarely detected with sparse distribution in the thyroid during anti-thyroid drug treatment [14]. In our study, there is selection bias to choose all Graves’ patients who eventually underwent thyroidectomy, but all hyperthyroid patients were pretreated with anti-thyroid drugs or inorganic potassium iodide, and the duration of Graves’ disease was not significantly different between the subjects and controls (Table 2).

A dense lymphoplasmacytic infiltration is the key morphologic feature of IgG4-related disease [1]. In Hashimoto’s thyroiditis, the degree of lymphoplasmacytic infiltration of IgG4 thyroiditis is significantly higher than that of non-IgG4 thyroiditis [5,15]. Based on the comprehensive diagnostic criteria of IgG4-related disease (more than 10 per high-power field and an IgG4/IgG rate of 40%) [1] or the cut-off value of IgG4 thyroiditis (more than 20 per high-power field and an IgG4/IgG rate of 30%) [5], almost half of Graves’ disease patients with diffuse lymphoplasmacytic infiltration had significantly higher levels of IgG4-positive plasma cells in the thyroid. Therefore, the histopathological finding of diffuse lymphoplasmacytic infiltration is atypical in Graves’ disease but indispensable to detect high levels of IgG4-positive plasma cells in the thyroid.

In our study, no persistent hyperthyroid patients with Graves’ disease who had high levels of IgG4-positive plasma cells in diffuse lymphoplasmacytic infiltration showed concomitant fibrosis or obliterative phlebitis (Table 1). The diagnostic criteria for IgG4-related disease place much importance on the morphological appearance on biopsy or surgical specimens including concomitant findings of stromal fibrosis along with IgG4-positive plasma cells [1]. Meanwhile, there are subtle variations of histopathological characteristics among some organs such that obliterative phlebitis is always present in the pancreas and submandibular glands but is observed much less often in the lacrimal glands [16]. In the thyroid gland, all patients with IgG4 thyroiditis present with stromal fibrosis along with abundant IgG4-positive plasma cells to a varying degree [5,15,17] but rarely with obliterative phlebitis [15]. Although it is uncertain whether stromal fibrosis or obliterative phlebitis coexists later in the presence of intense infiltration of IgG4-positive plasma cells, the present histopathological findings of Graves’ patients with persistent hyperthyroidism are unlikely to be those of IgG4 thyroiditis or IgG4-related disease.

While persistently hyperthyroid patients with Graves’ disease presented with no association with IgG4 thyroiditis in our study, 1 Graves’ patient (case 1) who rapidly developed hypothyroidism with a large goiter showed marked stromal fibrosis along with IgG4-positive plasma cells in the thyroid, which is consistent with IgG4 thyroiditis [6]. It is well-known that treatment with radioactive iodine is a factor causing fibrotic change in the thyroid and leading to hypothyroidism [18]. No subjects had a history of radioiodine treatment throughout their clinical courses. Other reports also described patients who rapidly developed hypothyroidism with a large goiter among Graves’ patients with high levels of IgG4 in serum or thyroid tissue [7,8]. One clinical characteristic they have in common is diffuse hypoechogenicity based on thyroid ultrasonographic evaluation [6–8], while all patients with persistent hyperthyroidism showed a coarse texture (Table 2). Diffuse hypoechogenicity reflects intense lymphoplasmacytic infiltration and fibrosis in the thyroid gland and is also a sign indicating severe follicular degeneration and hypothyroidism [19,20]. In several histologic subtypes of Hashimoto’s thyroiditis, a fibrosis variant and IgG4 thyroiditis show marked similarities and overlap to a large extent [15]. Both variants tend to show marked hypothyroidism due to developing follicular degeneration with severe stromal fibrosis and frequently show diffuse hypoechogenicity in the thyroid. Therefore, it may be more critical for the presence of high-grade fibrosis rather than IgG4-positive plasma cells alone to develop hypothyroidism in Graves’ disease.

In conclusion, persistent hyperthyroid patients with Graves’ disease who show the intense infiltration of IgG4-positive plasma cells in the thyroid manifest no histopathological association with IgG4 thyroiditis or IgG4-related disease. However, some Graves’ patients who rapidly develop hypothyroidism with high levels of IgG4 in serum or tissue and a diffuse hypoechoic structure by ultrasound are likely to present with histopathological compatibility.

## Supporting Information

S1 Table The prevalence of Graves’ ophthalmopathy in subjects and controls. Graves’ ophthalmopathy (GO) was diagnosed based on more than 3 by clinical active score and/or CT images such as extraocular muscles enlargement or increased orbital fat in clinical records.
(DOCX)
