# Supplementary material for: Graves’ Disease Patients with Persistent Hyperthyroidism and Diffuse Lymphoplasmacytic Infiltration in the Thyroid Show No Histopathological Compatibility with IgG4-Related Disease
Source: PLoS One. 2015 Jul 28;10(7):e0134143. doi: 10.1371/journal.pone.0134143 (PMC4517766; doi:10.1371/journal.pone.0134143)
Supplement: S1 Table — Graves’ ophthalmopathy (GO) was diagnosed based on more than 3 by clinical active score and/or CT images such as extraocular muscles enlargement or increased orbital fat in clinical records. (DOCX) [file pone.0134143.s001.docx]

S1 Table.

Graves’ ophthalmopathy Case number of 3 patients

- + with GO in subjects

Subjects (n=11) 8 3 (2), (3), (5)

Controls (n=80) 66 14
